# What will it take for the Global Plan priority countries in Sub-Saharan Africa to eliminate mother-to-child transmission of HIV?

**DOI:** 10.1186/s12879-019-4393-5

**Published:** 2019-09-16

**Authors:** Ameena E. Goga, Thu-Ha Dinh, Shaffiq Essajee, Witness Chirinda, Anna Larsen, Mary Mogashoa, Debra Jackson, Mireille Cheyip, Nobubelo Ngandu, Surbhi Modi, Sanjana Bhardwaj, Esnat Chirwa, Yogan Pillay, Mary Mahy

**Affiliations:** 1 Health Systems Research Unit, South African Medical Research Council, Pretoria, 0001 South Africa; 2 HIV Prevention Research Unit, South African Medical Researh Council, Pretoria, South Africa; 3 Department of Paediatrics, University of Pretoria, Pretoria, 0001 South Africa; 4 Division of Global HIV and Tuberculosis, Center for Global Health, Centers for Disease Control and Prevention, Atlanta, GA 30329 USA; 5 Department of HIV/AIDS, World Health Organisation, 27, CH-1211 Geneva, Switzerland; 6 Centers for Disease Control and Prevention, Pretoria, 0001 South Africa; 7 Health section, United Nations Children’s Fund (UNICEF), New York, 10017 USA; 8 School of Public Health, University of the Western Cape, Cape Town, 7535 South Africa; 9 Health section, UNICEF South Africa, Pretoria, 0001 South Africa; 10 Gender and Health Research Unit, South African Medical Research Council, Pretoria, 0001 South Africa; 11 School of Public Health, University of Witwatersrand, Johannesburg, 2193 South Africa; 12 Chief Director HIV/AIDS, TB, MCWHN, National Department of Health, Pretoria, 0001 South Africa; 13 Strategic Information and Evaluation Department, UNAIDS, 1211 Geneva, Switzerland

## Abstract

**Background:**

The 2016 ‘Start Free, Stay Free, AIDS Free’ global agenda, builds on the 2011-2015 ‘Global Plan’. It prioritises 22 countries where 90% of the world’s HIV-positive pregnant women live and aims to eliminate vertical  transmission of HIV (EMTCT) and to keep mothers alive. By 2019, no Global Plan priority country had achieved EMTCT; however, 11 non-priority countries had. This paper synthesises the characteristics of the first four countries validated for EMTCT, and of the 21 Global Plan priority countries located in Sub-Saharan Africa (SSA). We consider what drives vertical transmission of HIV (MTCT) in the 21 SSA Global Plan priority countries.

**Methods:**

A literature review, using PubMed, Science direct and the google search engine was conducted to obtain global and national-level information on current HIV-related context and health system characteristics of the first four EMTCT-validated countries and the 21 SSA Global Plan priority countries. Data representing only one clinic, hospital or region were excluded. Additionally, key global experts working on EMTCT were contacted to obtain clarification on published data. We applied three theories (the World Health Organisation’s building blocks to strengthen health systems, van Olmen’s Health System Dynamics framework and Baral’s socio-ecological model for HIV risk) to understand and explain the differences between EMTCT-validated and non-validated countries. Additionally, structural equation modelling (SEM) and linear regression were used to explain associations between infant HIV exposure, access to antiretroviral therapy and two outcomes: (i) percent MTCT and (iii) number of new paediatric HIV infections per 100 000 live births (paediatric HIV case rate).

**Results:**

EMTCT-validated countries have lower HIV prevalence, less breastfeeding, fewer challenges around leadership, governance within the health sector or country, infrastructure and service delivery compared with Global Plan priority countries. Although by 2016 EMTCT-validated countries and Global Plan priority countries had adopted a public health approach to HIV prevention, recommending lifelong antiretroviral therapy (ART) for all HIV-positive pregnant and lactating women, EMCT-validated countries had also included contact tracing such as assisted partner notification, and had integrated maternal and child health (MCH) and sexual and reproductive health (SRH) services, with services for HIV infection, sexually transmitted infections, and viral hepatitis. Additionally, Global Plan priority countries have limited data on key SRH indicators such as unmet need for family planning, with variable coverage of antenatal care, HIV testing and triple antiretroviral therapy (ART) and very limited contact tracing. Structural equation modelling (SEM) and linear regression analysis demonstrated that ART access protects against percent MTCT (*p*<0.001); in simple linear regression it is 53% protective against percent MTCT. In contrast, SEM demonstrated that the case rate was driven by the number of HIV exposed infants (HEI) i.e. maternal HIV prevalence (*p*<0.001). In linear regression models, ART access alone explains only 17% of the case rate while HEI alone explains 81% of the case rate. In multiple regression, HEI and ART access accounts for 83% of the case rate, with HEI making the most contribution (coef. infant HIV exposure=82.8, 95% CI: 64.6, 101.1, *p*<0.001 vs coef. ART access=-3.0, 95% CI: -6.2, 0.3, *p*=0.074).

**Conclusion:**

Reducing infant HIV exposure, is critical to reducing the paediatric HIV case rate; increasing ART access is critical to reduce percent MTCT. Additionally, our study of four validated countries underscores the importance of contact tracing, strengthening programme monitoring, leadership and governance, as these are potentially-modifiable factors.

## Introduction

**Fig. 1:**
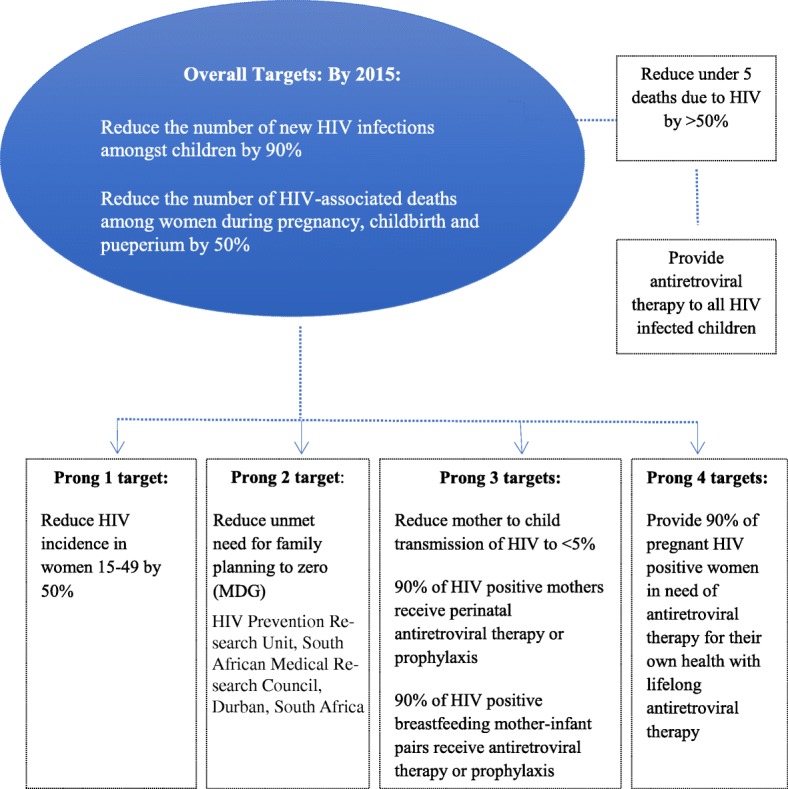
Targets for eliminating MTCT and keeping mothers alive

Since 2004, almost all countries globally have successfully reduced vertical transmission of HIV (MTCT), following a four-pronged approach developed by the World Health Organization (WHO) [1, 2] (Fig. 1). This includes preventing HIV amongst women of reproductive age; reducing unplanned pregnancy and unmet need for family planning; providing HIV testing and counselling and specific antiretroviral drugs during pregnancy, delivery and breastfeeding for women living with HIV and care, treatment and support to women living with HIV, and their families [3]. In 2011 the United Nations General Assembly high level meeting on AIDS launched the ‘Global Plan towards the Elimination of New HIV Infections Among Children by 2015 and Keeping Their Mothers Alive’ (Global Plan), committing to reduce the number of new HIV infections among children by 90% and the number of AIDS-related maternal deaths by 50%, by 2015 [2, 4, 5]. This Global Plan prioritized 22 countries (where 90% of the world’s HIV-positive pregnant women reside) for virtual elimination of MTCT as a public health problem (EMTCT), including one country in Asia (India), five countries in West Africa (Chad, Cameroon, Core d’Ivoire, Ghana, Nigeria), five in East Africa (Burundi, Ethiopia, Kenya, Uganda and United Republic of Tanzania), one in Central Africa (Democratic Republic of Congo) and ten in Southern Africa (Angola, Botswana, Lesotho, Malawi, Mozambique, Namibia, South Africa, eSwatini, Zambia and Zimbabwe) [2]. The commitment to EMTCT culminated in the policy transition to lifelong triple antiretroviral therapy (ART) for all pregnant and lactating women regardless of their CD4 cell count or disease staging, known as prevention of MTCT (PMTCT) Option B+ [6]. In 2016 the Start Free, Stay Free, AIDS Free global agenda was launched to build on the successes of the Global Plan [7]. It prioritises preventing new HIV infections among women and emphasizes retention of HIV-positive pregnant women and mothers on lifelong ART and early diagnosis and treatment for HIV-positive infants, children and adolescents.

**Fig. 2:**
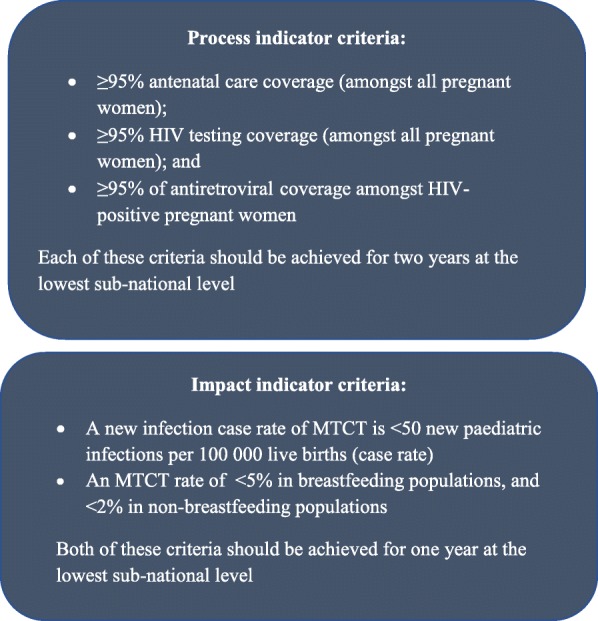
WHO criteria for validating elimination of HIV transmission from mother to child

PMTCT impact has traditionally been measured using MTCT at 6 weeks and 18 months postpartum. In 2014, the WHO, in collaboration with the Joint United Nations Programme on HIV/AIDS (UNAIDS), the United Nations Population Fund (UNFPA), and the United Nations Children’s Fund (UNICEF), released two impact and three process criteria to validate EMTCT [8, 9]. These should be achieved in at least one of the lowest subnational levels, e.g. a district for one (impact criteria) and two years (process criteria), to validate EMTCT (Fig. 2). The impact validation criteria focus on virtually eliminating MTCT as a public health problem, defined as percent MTCT less than 2% or 5% at final endpoint in non-breastfeeding or breastfeeding countries respectively, and reducing the case rate of new paediatric infections to 50 or less per 100 000 live births.

On June 30, 2015, the WHO announced validation of EMTCT in Cuba, a non-breastfeeding country [10]. Almost a year later Thailand, Armenia and Belarus were validated as having virtually eliminated MTCT as a public health problem [10, 11]. In 2017, Anguilla, Antigua & Barbuda, Bermuda, Cayman islands, Montserrat, St Christopher & Nevis, and in 2018, Malaysia met the EMTCT validation criteria (https://www.who.int/reproductivehealth/congenital-syphilis/WHO-validation-EMTCT/en/). In high-income countries, almost universal early ART coverage amongst HIV-positive pregnant women and avoiding breastfeeding have reduced MTCT risk to less than 1%. Despite substantial progress in low- and middle-income countries, which are mostly breastfeeding countries, the aggregate final MTCT at breastfeeding cessation is more than 5%, with case rates above the targeted 50 per 100 000 live births [6, 12, 13].

This paper synthesises the characteristics of the first four countries that met the EMTCT validation criteria (‘EMTCT-validated countries’), given that they have a longer history of EMTCT. We juxtapose these against the characteristics of the 21 Sub Saharan African (SSA) Global Plan priority countries that have not yet been EMTCT-validated. We conducted additional mediation and regression analyses to understand drivers of percentage MTCT and the EMTCT case rate, and consider what it will take for the 21 SSA Global Plan countries to achieve the EMTCT validation criteria.

## Methods

Global and national data published in English were sought to synthesise HIV- and PMTCT-related characteristics of EMTCT-validated countries and the 21 SSA Global Plan priority countries. PubMed, Google scholar and Science Direct were used to search for relevant peer- reviewed articles in English, using the terms MTCT, EMTCT and PMTCT effectiveness. As this paper mainly focuses on current status and is not a review of progress over many years, once a document with updated information on a particular indicator or topic was obtained, no additional searches were conducted for prior documents on that indicator. Documents or papers that only focused on a sub-national level such as one clinic, hospital or region were excluded as information from subnational settings were not relevant for this synthesis. Additionally, key individuals participating in global think tanks and expert groups were identified and contacted for relevant global or country-level reports, data, fact sheets and press releases on EMTCT or measuring PMTCT effectiveness. Although the EMTCT criteria specify that all criteria should be met in at least one of the lowest sub-national levels, this synthesis is restricted to national level, given the dearth of reliable data at subnational levels in SSA settings [14, 15]. We applied three theories, the WHO’s six building blocks to strengthen health systems, van Olmen’s Health System Dynamics framework and Baral’s socio-ecological model for HIV risk to understand and explain the differences between EMTCT validated and non-validated countries. The WHO theory states that strengthening six health system building blocks, namely (i) leadership and governance, (ii) health care financing, (iii) the healthcare workforce, (iv) medical products and technologies, (v) information and research and (vi) service delivery improves access, coverage, quality and safety of interventions, resulting in improved outcomes including equity, responsiveness and efficiency [16]. The van Olmen Health Systems Dynamics framework recognizes the existence of an overarching context within which health systems function, as well as the importance of leadership and governance, service delivery, resources (infrastructure, human resources, finances and knowledge and information) and population characteristics on goals and health outcomes [17]. Baral’s social ecological model acknowledges rings of influence on HIV risk, beginning at the individual level, expanding to social and sexual networks, community, public policy and HIV epidemic stage [18]. We considered these three models because they each adopt a different approach, ranging from system-specific [16], system within a context [17] to individual within a system and context [18]. We integrated the information from these theories to compare and understand the EMTCT-validated and SSA Global Plan priority countries, and consider what it will take to achieve EMTCT in the latter.

We used both linear regression and Structural Equation modelling (SEM) to estimate the contribution of infant HIV exposure (HEI) and ART access to percent MTCT (%MTCT) and the paediatric case rate (case-rate). The outcome case-rate was defined as the number of new paediatric HIV cases per 100 000 births, and the outcome %MTCT was percentage HIV transmission (numerator: number of new HIV infections amongst infants born to HIV infected mothers; denominator: number of HIV infected mothers), both assessed for 2017. The main exposure for both models was infant HIV exposure (HEI), defined as (number of HIV-positive pregnant women in 2017 *100)/total number of births in 2017. Due to limitations of sample size (number of countries), we reduced the number of variables in the models by creating an “ART-score”. This was defined as: ART-score = total access to treatment = ART access = % of HIV-positive on treatment (general population) + % HIV-suppressed (general population) + % of pregnant women on ART. Prior to deriving the additive score, we checked for internal consistency in the 3 ART-items (Cronbanch’s alpha=0.916) and also performed a confirmatory factor analysis to check how well the three items were loading on a single factor (root mean square error of approximation (RMSEA)<0.05, Tucker and Lewis Index (TLI)>0.95, comparative fit index (CFI)>0.95, coefficient of determination (CD)=0.94). We followed published rules of minimum levels of the fit indices, which specify minimum requirement for model acceptance for RMSEA values must be less than 0.06 and for CFIs and TLIs as 0.90 [19]. We also performed confirmatory factor analysis of the overall Structural Equation Model to check how well the model fitted the data (RSMEA<0.05, TLI, CFI>0.95, CD=0.88) and used the Sobel test to test for the significance of the mediating factor in both the structural equation model and the linear regression model [20]. For the linear regression model, we used the ‘product of coefficient’ method to test the mediation effects [20].

## Results

There is a stark contextual difference between EMTCT-validated countries and the 21 SSA Global Plan priority countries. These are presented within the three concepts referred to in the methods.

### Dynamic system within a context

Apart from the health system differences between the EMTCT-validated and 21 SSA Global Plan priority countries, their general HIV contexts differ considerably: the former have substantially lower HIV incidence and prevalence than SSA Global Plan priority countries (Table 1). Although EMTCT-validated countries have unknown unmet need for family planning, and low levels of HIV suppression, they have a low HIV incidence, and few people living with HIV (Table 1). Furthermore, the HIV epidemic is concentrated in injecting drug users (Belarus and Armenia) and men who have sex with men (Cuba and Thailand), rather than women of reproductive age, although the Armenian epidemic is increasingly becoming heterosexual [21–23]. Consequently, the number of HIV-positive pregnant women is low in EMTCT-validated countries with antenatal HIV prevalence at less than 1.3% across all four countries. The number of people living with HIV ranges from approximately 3 400 in Armenia to approximately 440 000 in Thailand [24]. Of these four countries, Thailand has the largest HIV epidemic, close in absolute numbers to Botswana, Ghana, Cote d’ Ivoire, Angola and the DRC (Table 1). In contrast, the 21 SSA Global Plan priority countries are mainly low- or middle-income with an HIV epidemic driven by heterosexual transmission, and numbers of people living with HIV ranging from 65 000 in Burundi to 7800 000 in South Africa (Table 1). Between 2009 and 2015, HIV incidence amongst women in Global Plan priority countries declined by 5%, rather than the 50% target and there were 4.5 million (3.8 milion-5.4 million) newly-infected women of reproductive age in Global Plan priority countries during this period [25]. South Africa added the largest number of new HIV infections in this group (1.2 million) followed by Nigeria (770 000) and Uganda (350 000)[25] Notwithstanding these challenges, between 2009 and 2015, the number of children newly infected with HIV declined by between 21% and 86% in the 21 SSA Global Plan priority countries: ten countries have reduced MTCT by >66% and seven by >70%; moreover, collectively these countries have reduced new paediatric HIV infections from 270 000 (230000-330000) in 2009 to 110 000 (78000-150 000) in 2015 [25, 26]. This is a significant improvement compared to the 24% reduction measured between 2000 and 2008 [25]. Most of the progress in PMTCT in the 21 SSA Global Plan priority countries occurred during the last five years; of the 1.4 million new HIV infections amongst children averted since 2000, 1.2 million were averted between 2009 and 2015 [25]. According to UNAIDS 2017 estimates, the final MTCT rate (i.e. measured at the end of breastfeeding), ranged from 5% in Botswana to 26% in Angola (Table 2) [3]. However, because HIV prevalence remains high, the paediatric HIV case rate, calculated as antenatal HIV prevalence multiplied by the %MTCT rate multiplied by 100 000 remains above the targeted 50 per 100 000 live births in many countries, including South Africa (where final %MTCT is estimated as 5.3%) (Table 2). This illustrates the role of maternal  HIV burden on meeting EMTCT targets.

**Table 1:** Characteristics of EMTCT validated countries and SSA Global Plan priority countries by December 2016

	New HIV^*^ infections Male+Female	HIV Incidence per 1000 ^*^ Male+Female	HIV population^*^ Male+Female	1st 90: %PLHIV who know their status ^*^(Target 90%)	Recommended CD4 for treatment initiation^╞^	2^nd^ 90: - Percent of People living with HIV receiving ART (Target 81%)^*^	3rd 90 :% viral suppression among all PLHIV (Target 73%)^*^	PMTCT coverage (Effective regimen 2010-2017)^*^	New HIV infections (15-49) Female (2017)^*^	Criminialisation of non-disclosure of HIV status^╢╞^	Women 15-49 who have their demands for family planning satisfied^╞^	Coverage of 4 antenatal visits during pregnancy % (year of data) ^╞^
Armenia	180 (120-230)	0.06 (0.04-0.08)	3400 (2800-4300)	66 (55-82)	≤500 cells/mm^3^	45 (37-55)	38 (31-47)	...	43 (70-61)	Yes	Could not find data	96% (2015-16)
Belarus	2400 (1500-3900)	0.27 (0.17-0.44)	24000 (18000-33000)	79 (58-108)	Treat all	46 (34-63)	30 (22-41)	92 (63-130)	798 (572-656)	Yes		100% (2012)
Cuba	1800 (1500-2200)	0.17 (0.14-0.19)	30000 (26000-33000)	80 (70-90)	≤500 cells/mm^3^	66 (58-75)	43 (38-49)	113 (93-128)	497 (569-600)	Yes		98% (2014)
Thailand	...	...	440000 (390000-510000)	98 (86-113)	Treat all	72 (63-83)	62 (55-72)	96 (79-115)		No		93% (2012)
Angola	27000 (20000-37000)	0.99 (0.73-1.36)	310000 (260000-360000)	...	Treat all	26 (22-30)		34 (24-42)	13,353 (13332-13737)	unknown	unknown	No data
Botswana	14000 (10000-16000)	7.47 (5.24-8.94)	380000 (340000-410000)	86 (78-93)	Treat all	84 (77-91)	81 (74-88)	90 (79-100)	6,710 (6140-6304)	Yes	unknown	73% (2007)
Burundi	3100 (1800-4600)	0.29 (0.17-0.44)	78000 (63000-93000)	80 (64-94)	Treat all	77 (62-91)		85 (61-105)	1,385 (1034-1103)	Yes	32%^*^^	33% (2010)
Cameroon	28000 (19000-36000)	1.24 (0.83-1.58)	510000 (430000-600000)	71 (59-82)	Treat all	49 (41-57)		77 (60-93)	13,828 (16118-17108)	Unknown	24%^*╢^	59% (2014)
Chad	5800 3600-7700)	0.41 (0.26-0.54)	110000 (94000-140000)	...	≤500 cells/mm^3^	45 (37-54)		68 (52-83)	2,500 (2510-2407)	Unknown	28%^*╧^	31% (2013-15)
Cote d’ Ivoire	30000 (13000-56000)	1.29 (0.55-2.43)	500000 (350000-690000)	54 (38-75)	Treat all	46 (32-63)	35 (25-49)	70 (45-103)	12,211	Yes	32.2%	44% (2011-12)
Democratic Republic of the Congo	15000 (8200-23000)	0.17 (0,09-0.27)	390000 (310000-470000)	59 (46-71)	Treat all	55 (43-66)		59 (44-74)	7,109 (10367-9237)	unknown	28%^*└^	48% (2011-12)
Eswatini	7000 (6200-7900)	8.02 (7.07-9.17)	210000 (190000-220000)	90 (83-97)	Treat all	85 (78-91)	74 (68-79)	90 (76-99)	3,362	No	15%^*║^	76% (2014)
Ethiopia	16000 (7200-28000)	0.17 (0.08-0.31)	610000 (470000-780000)	73 (56-94)	Treat all	71 (55-91)	32 (25-41)	59 (39-78)	6,229	Yes	60.5%	32% (2016)
Ghana	19000 (15000-24000)	0.68 (0.54-0.86)	310000 (260000-370000)	...	Treat all	40 (33-47)		66 (49-79)	9,771	Yes	33%^*└^	87% (2014)
Kenya	53000 (31000-86000)	1.21 (0.7-1)	1500000 (1300000-1800000)	...	Treat all	75 (63-89)	63 (53-74)	76 (58-92)	25,599	Unknown	80% (65-95%)	58% (2014)
Lesotho	15000 (13000-17000)	9.07 (7.66-10.37)	320000 (290000-340000)	80 (74-85)	Treat all	74 (68-78)	68 (63-72)	90 (76-99)	6,969	No	78.9%	76% (2014)
Malawi	39000 (35000-51000)	2.39 (2.11-3.18)	1000000 (980000-1100000)	90 (84-97)	Treat all	71 (66-77)	61 (58-67)	92 (75-105)	18,320	No	73.9%	51% (2015-16)
Mozambique	130000 (92000-170000)	4.75 (3.43-6.55)	2100000 (1800000-2500000)	59 (49-70)	Treat all	54 (45-65)		86 (64-109)	59,473	No	29%^*╢^	51% (2011)
Namibia	7400 5900-8400)	3.49 (2.73-4.01)	200000 (180000-210000)	90 (84-97)	Treat all	84 (79-91)	74 (69-80)	124 (101-139)	3,672	No	80.4%	63% (2013)
Nigeria.					Treat all					No^*^	unknown	51% (2013)
South Africa	270000 240000-300000)	5.46 (4.86-6.21)	7200000 (6600000-7900000)	90 (82-98)	Treat all	61 (56-66)	47 (43-52)	106 (82-125)	136,327	No	14%^*╨^	87% (2008)
Uganda	50000 (42000-59000)	1.37 (1.15-1.64)	1300000 (1300000-1400000)	81 (76-86)	Treat all	72 (68-77)	56 (53-60)	112 (90-126)	23,414	Yes	43%	48% (2011)
United Republic of Tanzania	65000 (58000-74000)	1.36 (1.21-1.55)	1500000 (1300000-1600000)		Treat all	66 (58-73)	48 (43-53)	85 (68-99)	28,240	Yes	55.1%	51% (2015-16)
Zambia	48000 (41000-58000)	3.6 (2.77-4.16)	1100000 (1100000-1200000)		Treat all	75 (70-81)		92 (78-103)	22,397	No^a^	21%^*└^	56% (2013-14)
Zimbabwe	41000 (29000-52000)	3.08 (2.18-3.96)	1300000 (1200000-1500000)	85 (74-96)	Treat all	84 (74-95)		96 (77-109)	19,255	Yes	84.8%	76% (2015)

^*^data from UNAIDS Spectrum model 2018 ^╢^prosecutions exist based on general law ^╞^UNAIDS DATA 2017. Available from: http://www.comminit.com/unaids/content/unaids-data-2017

**Table 2:** Current paediatric HIV case rate in sub-Saharan African (SSA) Global Plan priority countries

	Total Births Male+Female	Mothers needing PMTCT	% PMTCT coverage (Effective regimen 2010-2017)	%MTCT rate at 6 weeks	Final %MTCT rate including breastfeeding period	New HIV infections (0-14)	Paediatric HIV case rate (new paediatric HIV infections/births * 100,000)	% change in new infections 2010 to 2017
Angola	1200000	21000 (15000-26000)	34 (24-42)	14.7 (13.3-16.2)	26.1 (24.4-27.8)	5500 (3700-7100)	455	18%
Botswana	50000	12000 (11000-14000)	90 (79-100)	2.8 (1.6-3.8)	5.0 (3.4-6.3)	610 (360-850)	1,214	-27%
Burundi	460000	4900 (3600-6100)	85 (61-105)	6.3 (4.4-8.6)	13.9 (10.9-17.6)	690 (400-1100)	150	-44%
Cameroon	840000	30000 (23000-36000)	77 (60-93)	8.7 (5.9-10.9)	15.1 (11.2-17.9)	4500 (2600-6300)	538	-36%
Chad	630000	7500 (5700-9200)	68 (52-83)	9.8 (6.5-11.8)	17.9 (13.1-20.5)	1300 (780-1900)	213	-32%
Cote dIvoire	870000	25000 (16000-36000)	70 (45-103)	7.8 (5.0-11.2)	15.5 (9.2-20.5)	3800 (1500-7500)	442	-43%
Democratic Republic of the Congo	3600000	23000 (17000-29000)	59 (44-74)	11.7 (7.2-14.5)	20.4 (14.2-24.0)	4800 (2500-7100)	134	-51%
Eswatini	30000	10000 (8700-11000)	90 (76-99)	2.7 (1.6-4.3)	8.3 (6.6-10.3)	850 (600-1200)	2,841	-54%
Ethiopia	3200000	26000 (17000-34000)	59 (39-78)	11.3 (7.2-16.3)	21.2 (14.9-26.3)	5500 (2600-8800)	172	-43%
Ghana	870000	18000 (14000-22000)	66 (49-79)	10.5 (7.1-12.6)	18.7 (14.7-21.3)	3400 (2000-4700)	391	-31%
Kenya	1400000	69000 (53000-83000)	76 (58-92)	6.4 (4.1-9.1)	11.5 (8.5-15.4)	8000 (4600-13000)	573	-41%
Lesotho	53000	12000 (10000-14000)	90 (76-99)	4.6 (4.1-5.2)	11.3 (1.03-12.3)	1400 (1200-1600)	2,633	-35%
Malawi	640000	55000 (45000-62000)	92 (75-105)	3.0 (1.4-4.2)	8.9 (6.6-11.0)	4900 (3000-6700)	758	-61%
Mozambique	1200000	120000 (91000-160000)	86 (64-109)	6.8 (4.4-9.9)	14.4 (11.1-17.6)	18000 (10000-27000)	1,447	-44%
Namibia	68000	10000 (8100-11000)	124 (101-139)	1.7 (1.5-1.8)	6.0 (5.4-6.7)	600 (510-680)	878	-52%
Nigeria								
South Africa	1200000	250000 (190000-300000)	106 (82-125)	1.7 (1.6-3.5)	5.3 (4.7-7.1)	13000 (11000-22000)	1,146	-49%
Uganda	1600000	95000 (77000-110000)	112 (90-126)	4.2 (3.4-4.8)	7.9 (6.9-8.8)	7600 (6400-8600)	466	-57%
United Republic of Tanzania	1800000	94000 (75000-110000)	85 (68-99)	5.9 (3.8-7.8)	12.2 (9.3-14.4)	11000 (7200-15000)	629	-27%
Zambia	640000	71000 (60000-79000)	92 (78-103)	3.9 (3.3-4.4)	10.3 (8.6-11.9)	7300 (5400-9300)	1,137	-22%
Zimbabwe	510000	63000 (51000-73000)	96 (77-109)	2.4 (1.9-2.8)	6.7 (4.6-8.8)	4300 (2400-6300)	837	-64%

Calculated transmission rates, case rate and % change are based on unrounded numbers.

Source: UNAIDS 2018 estimates: Mary Mahy. Available at www.aidsinfo.unaids.org *refers to multiplied by

### System-specific factors relating to the six health system building blocks

In EMTCT-validated countries, the introduction of lifelong ART for pregnant and lactating women, synonymous with universal test and treat, occurred between 2011 (Cuba) and 2016 (Thailand), and coverage had expanded to more than 90% by 2016 [10, 11]. In all EMTCT-validated countries, integration between maternal and child health (MCH) and sexual and reproductive health (SRH) services and services for HIV infection, sexually transmitted infections, and viral hepatitis underpins all PMTCT-related activities [10, 11]. Additionally, HIV prevention services have a strong contact tracing component to prevent ongoing transmission and provide HIV services to those in need. For example, as early as 1983, the Cuban HIV prevention programme included contact tracing to identify and treat all people within the social networks of the HIV-positive person [27]. Furthermore, all health care, including HIV-related care, is free with an emphasis on prevention and community based care and empowerment of communities, resulting in high levels of uptake and adherence [28]. During antenatal care, more than ten visits are recommended with regular HIV testing and partner testing [29]. Additionally, the Cuban health system is constructed around the primary health care team; each team knows its catchment area, and conducts community-based needs assessments regularly and home visits to improve quality and coverage of care [28]. Similarly in Thailand, universal health coverage was achieved in 2002, with the introduction of short-term ART during breastfeeding, regardless of CD4 cell count in 2010, and lifelong ART for all pregnant and breastfeeding women in 2014 [30]. Partner testing is an integral part of HIV management in Thailand, and HIV testing rates in the general population  are more than 90% in three of the four EMTCT-validated countries. Over the past ten years, EMTCT-validated countries are less known for crises around leadership and governance, infrastructure, human resources and service delivery compared with Global Plan priority countries [30].

All of the 21 SSA Global Plan priority countries had adopted ART for pregnant and lactating women by 2016, and most had fully scaled up implementation by the end of 2017 [31]. Additionally, by 2016 most countries had adopted a ‘treat all’ approach for all persons living with HIV infection (Table 1). However, in only eight (38%) of the 21 SA Global plan priority countries, non-disclosure of HIV status is criminalised, compared with three of the four (75%)  EMTCT-validated countries (Table 1). Despite the commitment to introduce policies that enable HIV care and facilitate EMTCT, SSA Global Plan priority countries have large gaps in basic sexual and reproductive health services: unmet need for family planning is unknown in many SSA Global plan countries and variable among those that report data [3]; country-level coverage of four antenatal visits during pregnancy is variable, ranging from 31% to 87% in SSA Global plan countries, compared with 93-100% in EMTCT-validated countries (Table 1). Moreover, data demonstrate that in SSA Global Plan priority countries, few women receive 4 or more antenatal visits starting in the first trimester and even fewer receive eight or more antenatal visits starting in the first trimester, delaying PMTCT access [32, 33]. Despite these challenges, the 21 SSA Global Plan countries have adopted increasingly efficacious, evidence-based PMTCT policies, and pooled coverage of key PMTCT interventions (excluding single dose nevirapine) in the 21 Global Plan countries increased from 36% (32-41%) in 2009 to 80% (71-90%) in 2015 [25]. According to global reports, six of the priority countries (Botswana, Mozambique, Namibia, South Africa, eSwatini and Uganda) met the Global Plan goal of ensuring ≥90% ART coverage amongst pregnant women living with HIV [26]. However, viral load monitoring is sub-optimal in all countries and most countries are unable to provide disaggregated data to monitor pregnant and breastfeeding women. Furthermore, health systems within SSA Global Plan priority countries are plagued by challenges, and often crises with leadership and governance (in the health sector, and in general), infrastructure, healthcare financing, and availability of basic medical supplies, which are less prevalent in the EMTCT-validated countries [34]. These challenges weaken the health system and compromise service delivery, thus hampering further progress and EMTCT [16, 35].

### Individual level - stigma

At the individual level, surveys conducted in Thailand during 2010 and Belarus during 2013, amongst 233 and 370 people living with HIV, respectively, demonstrated the existence of self-stigma and external stigma from others: between 33% to 60% of interviewees reported self-stigma or being excluded from community or religious activities because of their HIV status [30, 31]. This seems similar to the self and external stigma described in SSA [36]. Thailand, Belarus and South Africa are taking steps to reduce stigma including advocacy, the involvement of religious leaders in stigma reduction training, removing negative portrayal of HIV in the media and individual support to reduce self-stigma [37]. It is noteworthy that the stigma in SSA Global Plan priority countries includes more females than in the EMTCT-validated countries, given the largely heterosexual nature of the epidemic and the larger numbers of HIV-positive people in SSA; thus gender issues and inequality are closely tied to ongoing stigma in SSA [37].

### Results of structural equation modelling and linear regression

SEM analysis demonstrated that in a model that includes ART access (ART score)  as a mediator in the relationship between HEI and %MTCT, ART score is protective against %MTCT (mediating effects coefficient: -0.41, 95% confidence interval (CI): -0.61,-0.21, *p*<0.001, Table 3) explaining 54% of the %MTCT. Linear regression corroborated these findings. In simple linear regression models, infant HIV exposure alone explains only 8% of the %MTCT and ART score explains 53% of the %MTCT. In the multivariable linear regression model assessing the relationship between HEI and %MTCT, with ART score as a mediator, ART score was associated with a lower %MTCT (mediating effects = -0.38, 95% CI: -0.64, -0.21, *p*<0.001, Table 3), and the multivariable regression model with ART score as a mediator explains 53% of the variation in %MTCT, Table 3 % (adjusted R^2^=0.53). In this multivariable model, the biggest contribution was made by ART score (coefficient -0.11 (95% CI: -0.16, -0.06, p<0.001). Results are different for the case rate: SEM demonstrated that ART score, as a mediator between HEI and paediatric case rate,  only protected against 13% of the paediatric case rate and this association was not significant (indirect effect= -10.6 95% CI -21.8, 1.8, p=0.077); the case rate was driven by infant HIV exposure (total effects= 71.6 95% CI 57,9, 85,3, *P*<0.001). Simple linear regression models show that ART access alone explains only 17% of the case rates while infant HIV exposure alone explains 81% of the case rates. In multiple regression infant HIV exposure and ART score account for 83% of the case rate, with infant HIV exposure making the most contribution (coef. infant HIV exposure=82.8, 95% CI= (64.6, 101.1), *p*<0.001 vs coef. ART score=-3.0, 95% CI=(-6.2, 0.3), *p*=0.074), Table 3.

**Table 3:** Structural equation modelling and mediation analysis to estimate the contribution of infant HIV exposure and ART score to %MTCT and the paediatric case rate

**Main outcome: %MTCT**					
a) *Structural equation model*					
	Path		Coef (95%CI)	Standardized coef	*p*-value
Direct effects	HEI → % MTCT		0.11 (-0.15, 0.37)	0.15	0.398
	ART_score → % MTCT		-0.11 (-0.15, 0.08)	-0.85	<0.001
	HEI → ART_score		3.64 (2.27, 5.02)	0.63	<0.001
Indirect (mediation) effect^b^	HEI → % MTCT		-0.41 (-0.61, -0.21)	-0.54	<0.001
Total effects	HEI → % MTCT		-0.30 (-0.54, -0.05)	-0.39	0.018
	ART_score → % MTCT		-0.11 (-0.15, -0.08)	-0.85	<0.001
	HEI → ART_score		3.65 (2.27, 5.02)	0.63	<0.001
b) *Linear Regression model*					
Model	independent variable	outcome	Coef (95% CI)	Standardized coef. (Adjusted R^2^)	*p*-value
Simple linear	HEI	% MTCT	-0.27 (-0.58, 0.04)	-0.35 (0.08)*	0.089
	ART_score	% MTCT	-0.10 (-0.14, -0.06)	-0.74 (0.53)*	<0.001
	HEI	ART_score	3.39 (1.41, 5.36)	0.60 (0.34)^*^	0.002
Multiple regression	HEI ART_score	% MTCT	0.11 (-0.17, 0.40)	0.15.^a^	0.418
		%MTCT	-0.11 (-0.16, -0.06)	-0.85 (0.53^a^)	<0.001
Mediated effects^b^	HEI	% MTCT	-0.38 (-0.64, -0.21)		<0.001
**Main Outcome: Paediatric case rate (case-rate)**					
a) *Structural equation model*					
Effect	Path		Coef (95%CI)	Standardized coef	*p*-value
Direct effects	HEI → Case_rate		82.2 (66.1,98.3)	1.03	<0.001
	ART_score → Case_rate		-2.9 (-5.7, -0.01)	-0.21	0.05
	HEI → ART_score		3.7 (2.0, 5.5)	0.64	<0.001
Indirect (mediation) effect^b^	HEI → Case_rate		-10.6 (-22.4, 1.2)	-0.13	0.077
Total effects	HEI → Case_rate		71.6 (57.9, 85.3)	0.90	<0.001
	ART_score → Case_rate		-2.9 (-5.7, -0.01)	-0.21	0.05
	HEI → ART_score		3.73 (2.0, 5.5)	0.64	<0.001
b) *Linear Regression model*					
Model	predictor	outcome	Coef (95% CI)	Standardized coef. (Adjusted R^2^)	*p*-value
Simple linear	HEI	Case_rate	72.8 (57.5, 88.2)	0.90 (0.81)^*^	<0.001
	ART_score	Case_rate	6.1 (0.92, 11.4)	0.45 (0.17)^*^	0.023
	HEI	ART_score	3.4 (1.4, 5.4)	0.60 (0.34)^*^	0.002
Multiple regression	HEI	Case_rate	82.8 (64.6, 101.1)	1.03 (0.83^a^)	<0.001
	ART_score	Case rate	-3.0 (-6.2, 0.31)	-0.20	0.074
Mediated effects^b^	HEI	Case_rate	-10.0 (-21.8, 1.8)		>0.05

HEI=Infant HIV exposure, case-rate= number of new paediatric HIV infections per 100 000 live births (paediatric HIV case rate), %MTCT= percent mother to child transmission of HIV, ART-score=% of HIV on treatment + % HIV-suppressed + % of pregnant women on ART.

^a^: Adjusted R^2^ for full multiple regression model ^b^ indicated mediated effects   *indicates proportion of outcome (out of 1) explained by this variable in simple linear regression

## Discussion

As described above, there are stark differences in the health systems and overall HIV epidemics in EMTCT-validated and the 21 SSA Global Plan priority countries. Noting these differences, we discuss what it would take for 21 SSA Global Plan priority countries to eliminate MTCT.

EMTCT requires prevention or early diagnosis of maternal HIV infection, immediate initiation of ART amongst HIV-positive women, post-natal infant prophylaxis and maternal viral suppression pre-conception, antenatally and during breastfeeding [6, 38]. Population-level data demonstrate that MTCT decreased to <1.2% when mothers initiated ART pre-conception, emphasising the importance of pre-conception test and treat strategies amongst women of reproductive age [39]. Meticulous patient tracking to reduce drop-out from care reduces MTCT but more importantly eliminating missed opportunities for diagnosing maternal HIV-infection leads to the largest drop in MTCT [40]. This is especially important given that data from South Africa show that incident HIV infections amongst pregnant and breastfeeding women may account for only 7% of all maternal HIV infections but 26% of MTCT [41]. There has been dramatic progress in reducing MTCT in SSA following the widespread adoption of a ‘universal test and treat’ approach for HIV-positive pregnant and breastfeeding women [6]. However, the ability of SSA countries to reduce MTCT further, particularly in breastfeeding countries will require substantial commitment including addressing the social and health system barriers to achieving maternal ART retention and viral suppression throughout pregnancy and the breastfeeding period (2 years or more in many countries). In SSA, where resources for health are stretched across multiple disease areas, scaling up routine viral load monitoring with urgent interventions for women who are not suppressed will require additional investments that optimise ART adherence and retention in care [42]. Enhanced psychosocial support and community engagement to reduce stigma will also be needed to complement health system improvements. Qualitative data from South Africa, a Global Plan priority country, demonstrated that lack of money to pay for transport, fear of an HIV-positive diagnosis, judgemental attitudes of health workers, lack of skilled staff and a lack of drugs and supplies hinder access to care [43]. Regardless of setting, stigma reduction and normalisation of HIV as a chronic disease will facilitate access to and uptake of care: A meta-analysis and systematic review published in 2017 illustrated that stigma reduction strategies have small effects in improving HIV knowledge and reducing negative attitudes towards people living with HIV; additionally, they are more effective amongst professionals, or if multiple sessions are conducted or if programmes are implemented in community settings [44].

In high HIV prevalence settings, dominated by heterosexual HIV transmission, the case rate could be lowered by reducing HIV incidence among women of reproductive age. [45] Antiretroviral use to prevent HIV acquisition in high risk HIV negative individuals, also known as pre-exposure prophylaxis or PrEP, has demonstrated effectiveness in at least two large-scale clinical trials, namely Partners PrEP and iPrEx, but there are no randomised trials data on its use in pregnant or lactating women [46, 47]. Partners PrEP enrolled heterosexual sero-discordant couples in Kenya and Uganda and demonstrated a 75% reduction in HIV acquisition overall, and a 90% reduction in HIV acquisition in participants with detectable drug in their blood.[47] The partners PrEP study documented 288 pregnancies exposed to PrEP during the first few weeks of pregnancy. [48] As per study protocol PrEP was stopped in these women. Infant follow-up during the first year of life demonstrated no statistical difference in adverse infant outcomes. Further study of PrEP during pregnancy and lactation are ongoing. Although PrEP use is supported amongst pregnant and lactating women whose HIV positive partners have unsuppressed viral load in Australia, Canada, France, New Zealand, the USA and UK, in SSA, only Kenya, Uganda and eSwatini support such use in sero-discordant couples. Research is needed to establish foetal safety, and optimal target group in high HIV prevalence settings where most pregnant women describe themselves as single.[49] Whilst these data are awaited other evidence-based HIV prevention strategies are needed to reduce HIV incidence in women of reproductive age. These include voluntary medical male circumcision, which is an effective intervention to reduce horizontal HIV transmission and offers protection against cervical cancer, chlamydia and syphilis; however, it offers little direct control over the risk process to vulnerable, disempowered women [50]. Similar to other researchers, we believe that multipronged holistic approaches addressing upstream structural, biomedical, legal, social, gender-related and health system drivers of HIV incidence are needed, in addition to biomedical interventions implemented at scale [51–53]. Structural interventions include sexual and reproductive education amongst adolescents, young and older women and men, family planning, and addressing social constructs of masculinity [51]. Such a holistic prevention framework, which includes supporting the economic advancement of women, can facilitate self-protection and HIV avoidance [51, 53]. One potential success story is the PEPFAR pioneering DREAMS (Determined, Resilient, Empowered, AIDS-free, Mentored and Safe) public-private partnership currently implemented in 63 districts within 10 African countries: DREAMS implements a core package that combines evidence-based approaches within the health sector, with those that address structural drivers that increase girls’ HIV risk, including poverty, gender inequality, sexual violence, and a lack of education, and achieved a 25% decline in new HIV diagnoses amongst girls and young women aged 15-24 between 2015 and November 2017 [54].

## Conclusions

Drawing from the characteristics of EMTCT-validated countries, we deduce that offering universal ART is just one important step towards EMTCT validation. Simultaneous health system strengthening to improve service delivery, client tracing, programme monitoring, leadership and governance, and social, educational and structural interventions to reduce HIV incidence amongst women of reproductive age and HIV-related stigma are critical, potentially modifiable contextual factors that determine success.

## Data Availability

Not applicable – all data are drawn from the reports in the public domain

## Abbreviations

ART: Triple antiretroviral therapy
EMTCT: Eliminating mother-to-child transmission of HIV
MTCT: Mother-to-child transmission or vertical transmission of HIV
PMTCT: Preventing mother-to-child transmission of HIV
SSA: Sub-Saharan Africa
STI: Sexually transmitted infections
UNAIDS: Joint United Nations Programme on HIV/AIDS
UNFPA: United Nations Population Fund
WHO: World Health Organisation
